# An integrative top-down and bottom-up qualitative model construction framework for exploration of biochemical systems

**DOI:** 10.1007/s00500-014-1467-6

**Published:** 2014-09-30

**Authors:** Zujian Wu, Wei Pang, George M. Coghill

**Affiliations:** 1 College of Information Science and Technology, Jinan University, Guangzhou, 510632 People’s Republic of China; 2 School of Natural and Computing Sciences, University of Aberdeen, Aberdeen, AB24 3UE Scotland, UK

**Keywords:** Evolution strategy, Simulated annealing, Qualitative model learning, Top-down and bottom-up modelling, Systems biology

## Abstract

Computational modelling of biochemical systems based on top-down and bottom-up approaches has been well studied over the last decade. In this research, after illustrating how to generate atomic components by a set of given reactants and two user pre-defined component patterns, we propose an integrative top-down and bottom-up modelling approach for stepwise qualitative exploration of interactions among reactants in biochemical systems. Evolution strategy is applied to the top-down modelling approach to compose models, and simulated annealing is employed in the bottom-up modelling approach to explore potential interactions based on models constructed from the top-down modelling process. Both the top-down and bottom-up approaches support stepwise modular addition or subtraction for the model evolution. Experimental results indicate that our modelling approach is feasible to learn the relationships among biochemical reactants qualitatively. In addition, hidden reactants of the target biochemical system can be obtained by generating complex reactants in corresponding composed models. Moreover, qualitatively learned models with inferred reactants and alternative topologies can be used for further web-lab experimental investigations by biologists of interest, which may result in a better understanding of the system.

## Introduction

The goal of understanding species behaviour and essential functions of a natural biochemical system can be achieved by obtaining information of individual parts and corresponding interactions within the system. Models can be constructed to represent the given systems, and these models can exhibit the same characteristics by simulations. In general, two different but complementary strategies can be applied to model biochemical systems: top-down and bottom-up approaches. Biological modelling tasks, including investigation of mechanisms and principles in the biological system, revealing underling cell functions of the system and formalising biological processes in cells to achieve a better understanding of the system, can be addressed by the top-down and bottom-up approaches.

From a modeller’s point of view, a given cellular system can be reduced systematically in a top-down manner until essential parts remain in a minimal cellular environment; while in a bottom-up approach, the whole or part of a target biological system can be composed from individually meaningful components. Therefore, during the modelling process, biochemical systems are simplified by the top-down modelling route, and atomic or prototypical biochemical units can be assembled by the bottom-up modelling route to achieve the goal of constructing the whole model. More details about the top-down and bottom-up approaches in the context of modelling biochemical systems are described in Bruggeman and Westerhoff (2007), which also discussed challenges in modelling biochemical systems and limitations of these two approaches.

In the top-down modelling approach, while a large biochemical system is decomposed to discover molecular mechanisms, correlations among concentrations of molecules can be discovered from these obtained mechanisms. More biochemical assumptions may be suggested and verified in further biochemical analysis and wet-lab experiments. In addition, studies on cell interactions can benefit from the top-down approach, in which large datasets are dealt with in a controllable manner and knowledge of biochemical system behaviour is obtained and described in a systematic manner. Furthermore, predictions of biological mechanisms (Taylor et al. 2003; Ihmels and Bergmann 2004) and functional processes (Tanay et al. 2004; Beyer et al. 2006) can be supported by the discovery of behavioural patterns in the system.

The availability of large omics data makes it possible for implementing the top-down modelling approach, which enables us to analyse the dynamics of the system at genomic level and address biochemical issues. For instance, metabolome, fluxome, transcriptome and/or proteome can all be improved and completed by the use of the top-down approach (Westerhoff and Palsson 2004). Moreover, structures of the molecular networks can be identified (Kholodenko et al. 2002; Vlad et al. 2004) and parameter values in gene networks can be determined (Moles et al. 2003; Kremling et al. 2004).

In the bottom-up modelling approach, functional patterns of a biochemical system may be discovered by integrating biochemical units into a whole complex biochemical system from scratch. These units describe interactions among species and contain relevant biochemical information, for instance, kinetic laws of biochemical reactions. In the bottom-up modelling process, interactions among a small group of components are composed to formulate a functional sub-system, such as enzymatic reactions. The synthetic sub-systems can also be composed to explore functional interactions in the target biochemical systems, which produces models to approximate and predict behaviour of the target systems—such behaviour data are obtained from literatures or experimental data. Therefore, topologies and kinetic rates associated with reactions of biochemical systems can be constructed in a stepwise bottom-up modelling manner.

Over the last decade, many biochemical pathways have been investigated by applying the bottom-up modelling strategy with the support of experimental validation. For instance, modelling of the downstream signalling network of the epidermal growth factor receptor (Kholodenko et al. 1999; Suenaga et al. 2004; Kiyatkin et al. 2006), the central carbon metabolism in *E. Coli* (Kremling et al. 2001; Schmid et al. 2004; Bettenbrock et al. 2006) and glycolysis in bloodstream from *Trypanosoma brucei* (Albert et al. 2005).

Computational modelling of biochemical systems aims to generate models representing target biochemical systems in terms of behaviour and interactions among biochemical components controlled by kinetic rates and concentrations of species. In the presence of abundant quantitative data and sufficient knowledge about the system, it is straightforward to employ sophisticated modelling approaches and tractable computational tools to quantitatively model such systems by fitting the kinetic parameters according to concentrations of measured species. However, when only incomplete knowledge and sparse, noisy data are available, it is essential to use qualitative model learning approaches, which qualitatively construct and analyse biochemical models (Steggles et al. 2007), from which observations of a given biological system can be explained by exploring the interactions among reactants in these models at a qualitative level.

Therefore, our research focuses on using systems biology (SB) approaches to modelling real-world biochemical pathways when there are only incomplete knowledge and qualitative data available. In particular, there exist imprecision, uncertainty, and approximation issues when modelling pathways as such in SB. These issues can be well addressed by soft computing approaches, as they are key topics in the field of soft computing. More specifically, it is still intractable for some conventional mathematical and analytical methods when they are applied to qualitatively inferring structures of complex pathways. So, in this research, we aim to employ well-established soft computing algorithms to tackle the aforementioned issues in SB and it is expected that satisfactory solutions can be obtained.

With regard to the availability of both top-down and bottom-up modelling approaches, in this research, we propose a framework which employs an integrative top-down and bottom-up approach to the evolutionary exploration of the biochemical model space. The top-down approach is applied to sub-models of a given complicated biochemical system, and it heuristically explores meaningful components in a stepwise manner. Then, the bottom-up approach is used to further develop obtained components from the top-down approach, discovering potential interactions among reactants in the target system.

In general, our stepwise integrative top-down and bottom-up qualitative model learning (QML) approach is briefly described as follows. The modelling of a biochemical pathway involves construction of a library to preserve the biochemical functional components according to user specifications. Evolution strategy (ES) (Schwefel 1965; Beyer and Schwefel 2002) is employed in the top-down route to perform efficient selection and composition of functional modules to evolve models seeds (those models with partial sub-models of target biochemical systems) toward complicated dynamic models. Simulated annealing (SA) (Kirkpatrick et al. 1983) is used to further develop components composed from the top-down approach, and it keeps to add and verify extra newly explored components. A set of qualitative states is then obtained from the explored models, with which qualitative experimental data of the target biochemical systems are compared. Thus, qualitative states of reactants in target biochemical systems are used to guide the model construction during the evolutionary modelling process. These developed and evolved models are finally used by biologists and modellers to confirm experimental results, verify hypotheses made upon the target biochemical system, and predict undiscovered biochemical processes with hidden components.

The motivation of employing ES and SA in this research is that they have been proven to be effective in similar problems in our previous work (Wu et al. 2012, 2014). ES and SA have been employed to solve problems of quantitatively modelling biochemical systems in terms of topology and kinetic rate constants. In this research, we focus on qualitative modelling of biochemical systems, which has a similar task to analyse and obtain the structure of a target biochemical system using qualitative states abstracted from numerical quantitative data.

Moreover, our previous work tackles the problems of composing models from atomic components by a hybrid ES and SA method. The performance of different ES–SA variants has been well studied for understanding the effectiveness of ES and SA algorithms when performing the heuristic search and computation modelling. So, we expect that they can also achieve good performance in the problem to be addressed in this research. In addition, at the current stage of the research, we focus on solving real-world biochemical system modelling issues by soft computing and metaheuristic approaches, and to start with we choose ES and SA. But we also point out that in the future, more soft computing approaches may be investigated for their suitability of our particular problems.

The rest of this paper is organised as follows. In Sect. 2, we present the integrative modelling approach by illustrating the top-down model composition and bottom-up model exploration methodologies, respectively, in which basic components and models are defined and described. Moreover, genetic operators are applied to evolve the models. In Sect. 3, we describe QML, including how to analyse qualitative states obtained from qualitative differential equations (QDEs) for model evaluation. Case studies and simulation results with analysis are reported in Sect. 4. Finally, Sect. 5 concludes the paper with discussions on future work.

## Integrative top-down and bottom-up modelling

In this section, definitions of components and models are first presented before evolutionary modelling of biochemical pathways is introduced. Components are basic building blocks in our stepwise qualitative modelling approach, and Petri nets (Murata 1989) are used to represent the structure of a component. Components are composed using a set of composition rules to form models which represent the target biochemical pathway. A model should at least consist of one component, which ensures a minimal model structure for further evolutionary model construction.

### Components and models

Biochemical components can be defined by Petri nets, as reported in our previous work (Wu et al. 2010, 2012). There are two patterns for instantiating components: binding and unbinding patterns, as shown in Eqs. (1) and (2).

1 $$\begin{aligned}&P_1+P_2 \mathop {\longrightarrow }\limits ^{k1} P_3\end{aligned}$$

2 $$\begin{aligned}&P_3 \mathop {\longrightarrow }\limits ^{k2} P_1+P_2 \end{aligned}$$

In Eq. (1), $P_1$ represents a reactant acting as a substrate; $P_2$ denotes a reactant acting as an enzyme, and $P_3$ ($P_3=P_1|P_2$) is a complex synthesised from $P_1$ and $P_2$ at the reaction kinetic rate of $k1$, which represents the speed of the synthetic process. Therefore, in this study, we use the symbol ‘$|$’ joining the labels of the two reactants to represent a complex.

In Eq. (2), $k2$ is a reaction kinetic rate constant for a disassociation process; complex $P_3$ is either disassociated to two reactants $P_1$ and $P_2$ which form the complex itself—an inverse process of the binding pattern in Eq. (1); or converted into a new product and an enzyme which is one of the reactants forming the complex $P_3$.

We take the binding pattern for instantiation of a component, in which two reactants are combined to form a complex reactant, and the unbinding pattern for instantiation of a component, in which a complex reactant is divided into two reactants.

For those complicated components consisting of more than three reactants, we can represent them by combining the components instantiated from these two patterns. Note that our current research focuses on qualitative modelling of biochemical pathways, therefore only the structures of the biochemical models are considered, and the kinetic rate constants of the models are not under our investigation. Thus, the rate constants are not associated on these components.

We briefly introduce the formation of components from two sets of reactant labels provided by the users: given a set of reactants as species $S_\mathrm{species}$ and another set of reactants as enzymes $S_\mathrm{enzymes}$, each element in $S_\mathrm{species}$ is selected in turn to be combined with each element in $S_\mathrm{enzymes}$ to produce a complex and a new reactant, based on the mass-action 1 (MA1) kinetic law (Breitling et al. 2008). For instance, species $A$ from $S_\mathrm{species}$ is combined with enzyme $E$ from $S_\mathrm{enzymes}$ to form a complex $A|E$ and a new reactant $AP$. A synthetic enzymatic reaction is shown in Eq. (3). In this equation, the symbol ‘$\rightleftharpoons$’ indicates that the reaction is reversible; the symbol ‘$\rightarrow$’ presents a non-reversible reaction; the symbol ‘$|$’ indicates that a complex reactant is generated from the two reactants (as described before); and the letter $P$ after the species label $A$ means a new product generated from $A$.

3 $$\begin{aligned} A + E \rightleftharpoons A|E \rightarrow AP + E \end{aligned}$$

Therefore, three atomic components can be obtained from an enzymatic reaction: ‘$A + E \rightarrow A|E$’, ‘$A|E \rightarrow A + E$’ and ‘$A|E \rightarrow AP + E$’.

**Fig. 1:**
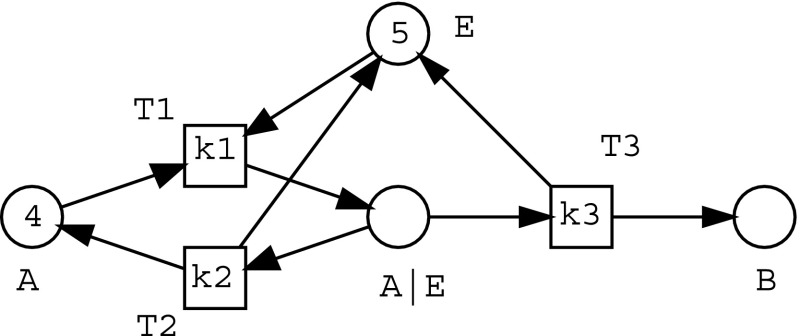
A graphical presentation of a Petri net model for an enzymatic reaction consisting of three components

The components generated from the sets of reactants given by a user become parts of a model under construction in this research. A model consists of many components, which are connected with each other by merging the same ‘nodes’ (Places) in a Petri net (Wu et al. 2012). Figure 1 shows a Petri net model of an enzymatic reaction which consists of three components connected with each other by merging the same reactants. There should be at least one component in a model during the stepwise evolutionary model learning process, because the additions of other components cannot be performed on a model without anything.

### Top-down model composition

In the top-down modelling approach, each individual in the population maintained for evolutionary construction is a Petri net model. At each generation during the evolutionary process, the topology of each Petri net model is evolved through the application of genetic addition and subtraction operators. Individual model seeds at the initial evolutionary stage are connected sub-models of the target biochemical system. An evolutionary algorithm $(\mu +\lambda )-\mathrm{ES}$ (Beyer and Schwefel 2002) is employed to iteratively stepwise assemble components to develop models. To test our stepwise evolutionary modelling approach in a simplest scenario, we choose to generate offsprings by a simple $(1+1)-\mathrm{ES}$. Further advanced $(\mu +\lambda )-\mathrm{ES}$ will be performed and investigated for the future study of the stepwise evolutionary modelling framework.
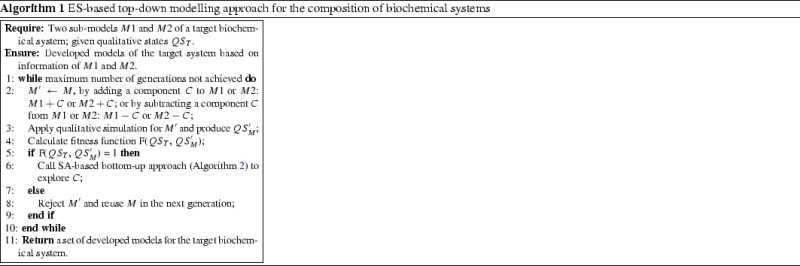


Algorithm 1 shows the pseudo-code of using ES to perform top-down modelling of biochemical systems. There are two sub-models $M1$ and $M2$ of a target biochemical system. These sub-models can be obtained from literature or knowledge of wet-lab by experiments. A composed model $M'$ can be generated by applying addition or subtraction operators (Wu et al. 2012) to $M1$ and $M2$. Model $M'$ is simulated and evaluated qualitatively by employing the qualitative simulator JMorven (Bruce and Coghill 2005), which can generate a set of qualitative states $QS_M'$ for comparison with $QS_T$ in a fitness function F (as shown in Steps 3 and 4 of Algorithm 1). The calculation of the fitness function will be described later in Sect. 3. If the fitness value is equal to 1 (the range of fitness value is from 0 to 1, and the bigger the better), an SA-based bottom-up approach in Algorithm 2 will be used to explore potential components by trails of component additions. Otherwise, the mutated $M'$ is rejected and the mutation is performed in the next generation. The reason of calling Algorithm 2 to explore added component is that the model with the fitness value being equal to 1 cannot be further distinguished by Eqs. (4)–(6) on the trails of component additions. Model exploration requires to perform estimation of constructed models in a probabilistic manner. The top-down modelling is terminated if the ES stop conditions are satisfied. In this situation, a set of final best models will be output with explored components for the given biochemical system.

### Bottom-up model exploration

Simulated annealing is one of the heuristic algorithms for searching the global optimal solution in a very large solution space, and it can avoid local optima. As shown in our previous work (Wu et al. 2010), topologies of biochemical models represented by Petri nets can be piecewise constructed and explored by employing SA. In this research, the bottom-up approach uses SA to explore important components obtained from the top-down approach, in which ES is used to perform model composition.
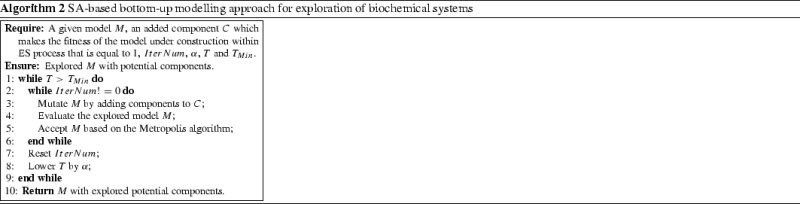


Algorithm 2 shows the pseudo-code of the bottom-up exploration of possible components in a given model. There are a given model $M$ and an added component $C$ which makes the fitness of the model under construction in the ES process is equal to 1. Possible components are selected from the component library and added to $C$ for model exploration, considering improvement of the Bayesian score, which will be described in Sect. 3. The parameter $t$ is the current SA system temperature ($t=T$), and $IterNum$ is the number of iterations at each system temperature. The mutated $M$ with the explored component $C$ is evaluated at each iteration by calculating the Bayesian scores of the mutants of $M$.

The evaluated model $M$ with explored components is accepted or rejected according to a classical Metropolis mechanism (1953). Accepted $M$ is preserved as a new start seed for the next run of model explorations. Model $M$ with different explored components is mutated heuristically at different SA system temperatures by a cooling rate $\alpha$. The whole optimization process will stop when the system temperature reaches the minimum temperature $T_{Min}$.

Note that, due to the probabilistic and random nature of SA (Anily and Federgruen 1987), a mutated model $M$ with a poor estimated fitness value could be generated and accepted.

### An integrative modelling approach

**Fig. 2:**
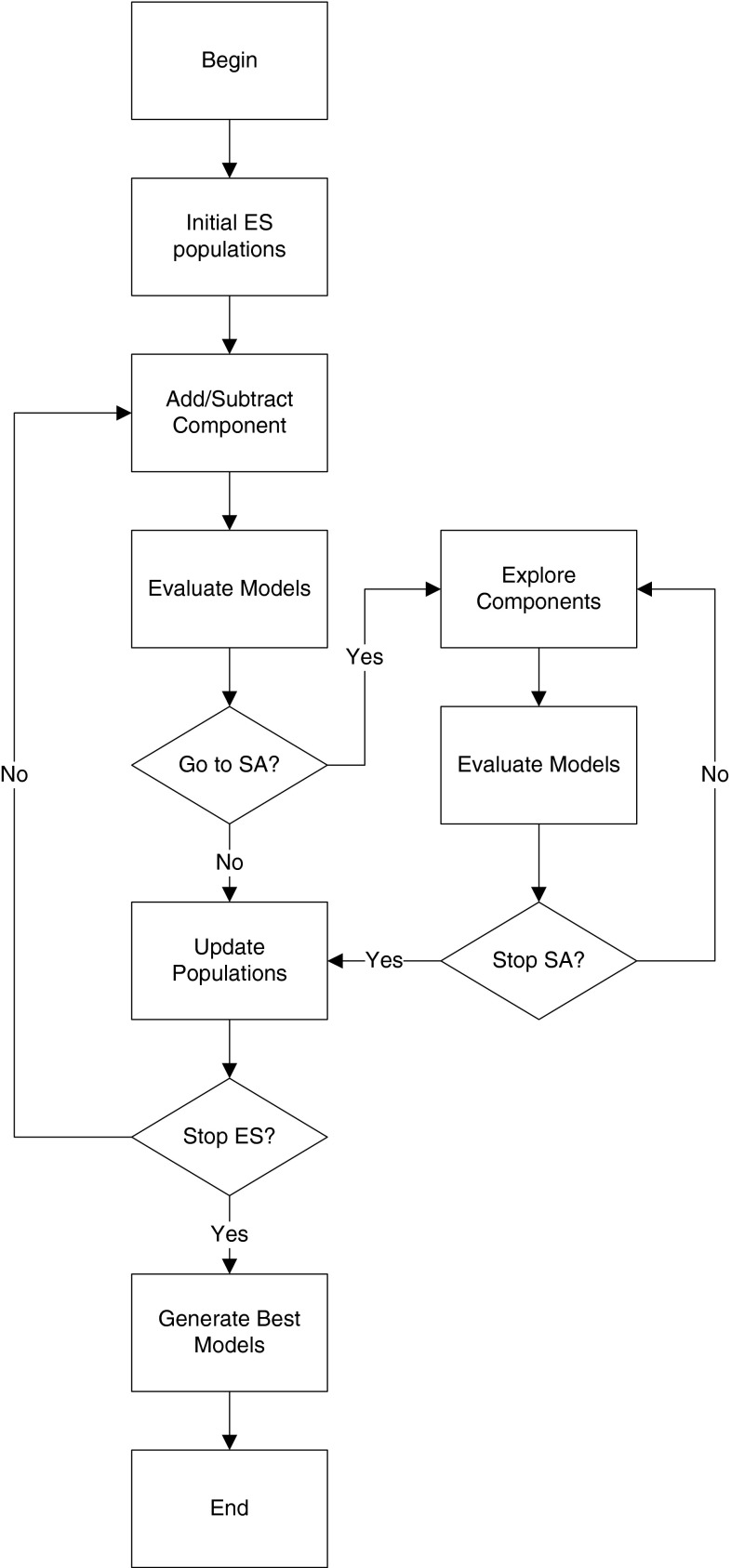
A general illustration of the integrative modelling process based on ES and SA in the top-down and bottom-up routes, respectively

In this research, we propose to integrate two metaheuristic algorithms (ES and SA) within two different modelling routes (top-down and bottom-up), respectively, for constructing models. ES is a population-based metaheuristic algorithm and it is good at generating alternative solutions probabilistically. We utilise ES to tackle the model composition in our proposed integrative model construction process. SA is a single-solution based metaheuristic algorithm, which obtains optimal solutions by global search. Thus, we employ SA to explore potential interactions between reactants in a model under construction. A flowchart in Fig. 2 illustrates the integrative model development and exploration by switching model compositions between ES and SA during the modelling process.

These two metaheuristic algorithms are used to solve modelling issues in a collective manner. While potential and meaningful components are added to models under construction in the top-down modelling route, more detailed interactions among reactants could be explored using the given component through the bottom-up modelling route.

## Qualitative model learning

Qualitative information rather than quantitative information is employed in qualitative reasoning (QR) (Forbus 1996; Kuipers 1994) to achieve the aims of modelling real-world problems. The main task of QR is to automatically infer continuous aspects of a given complex system in terms of space, time and quantity. In QR research, complex dynamic systems are described by qualitative values, for instance, *high*, *medium*, *low*, *zero*, *positive* and *negative*, instead of using precise numerical quantities. Qualitative representation and reasoning methodologies enable the behaviour of given complex systems to be reasoned in silico in the presence of incomplete background knowledge and imperfect data.

Qualitative model learning is a sub-branch of QR, which involves automatic extraction of QDE models of dynamic systems from available data (Pang and Coghill 2010a). QML systems have been well studied and several QML systems have been developed, for instance, MISQ (Richards et al. 1992), GENMODEL (Hau and Coiera 1993), QSI (Say and Kuru 1996), QOPH (Coghill et al. 2002), ILP-QSI (Coghill et al. 2008), and the most recent QML-Morven framework (Pang 2009; Pang and Coghill 2014). As a complementary approach to quantitative modelling, QML works well in reasoning dynamic systems, especially when there are only noisy and sparse data available. QML can infer and suggest plausible qualitative models of a target dynamic system. These plausible models could be further investigated and verified by quantitative modelling approaches.

### Qualitative states

A given dynamic system can be described at a qualitative level, and its important behavioural properties are captured by a set of qualitative states and possible transitions between these states. A qualitative state is a complete assignment of qualitative values to all variables in the system and considered as a ‘snapshot’ of the system. The dynamic system under investigation could demonstrate such possible qualitative states and a correct model built for the system should reproduce these qualitative states (and only these states if all variables are known).

Table 1 shows a set of qualitative states derived from a qualitative model. Each row in this table represents an individual qualitative state. For each variable, its magnitude and rate of change of the current state are illustrated by qualitative signs: *pos* (positive), *zer* (zero) and *neg* (negative). For example, if a qualitative value of a variable *A* is $\langle \mathrm{zer}, \mathrm{pos}\rangle$, this means the magnitude of *A* is zero and the rate of change is positive, which indicates that the value of *A* is increasing.

**Table 1:** A set of qualitative states

State ID	A	AP	B	BP
1	$\langle$zer , pos$\rangle$	$\langle$pos , neg$\rangle$	$\langle$pos , neg$\rangle$	$\langle$pos , neg$\rangle$
2	$\langle$pos , pos$\rangle$	$\langle$pos , pos$\rangle$	$\langle$zer , pos$\rangle$	$\langle$pos , neg$\rangle$
3	$\langle$pos , zer$\rangle$	$\langle$pos , zer$\rangle$	$\langle$pos , neg$\rangle$	$\langle$pos , neg$\rangle$
4	$\langle$pos , pos$\rangle$	$\langle$pos , pos$\rangle$	$\langle$pos , neg$\rangle$	$\langle$pos , neg$\rangle$
5	$\langle$pos , neg$\rangle$	$\langle$zer , pos$\rangle$	$\langle$pos , neg$\rangle$	$\langle$pos , neg$\rangle$
6	$\langle$zer , pos$\rangle$	$\langle$pos , neg$\rangle$	$\langle$pos , zer$\rangle$	$\langle$pos , neg$\rangle$
7	$\langle$pos , zer$\rangle$	$\langle$pos , neg$\rangle$	$\langle$pos , zer$\rangle$	$\langle$pos , neg$\rangle$
8	$\langle$pos , neg$\rangle$	$\langle$pos , zer$\rangle$	$\langle$pos , zer$\rangle$	$\langle$pos , neg$\rangle$
9	$\langle$pos , zer$\rangle$	$\langle$pos , zer$\rangle$	$\langle$pos , zer$\rangle$	$\langle$pos , neg$\rangle$
10	$\langle$pos , neg$\rangle$	$\langle$pos , pos$\rangle$	$\langle$pos , zer$\rangle$	$\langle$pos , neg$\rangle$
11	$\langle$zer , zer$\rangle$	$\langle$zer , zer$\rangle$	$\langle$zer , zer$\rangle$	$\langle$zer , zer$\rangle$
12	$\langle$pos , pos$\rangle$	$\langle$pos , zer$\rangle$	$\langle$zer , zer$\rangle$	$\langle$zer , zer$\rangle$
13	$\langle$pos , pos$\rangle$	$\langle$pos , zer$\rangle$	$\langle$pos , zer$\rangle$	$\langle$pos , zer$\rangle$
14	$\langle$pos , pos$\rangle$	$\langle$pos , neg$\rangle$	$\langle$pos , pos$\rangle$	$\langle$pos , zer$\rangle$

For the legal transitions among the states, transition rules [for instance, rules in QSIM (Kuipers 1994)] are employed to calculate them. A sequence of qualitative states form a *qualitative behaviour*, and the terminal states of the qualitative behaviour are often equilibrium states, in which all variables remain constant (Pang and Coghill 2011).

### Qualitative differential equations

One of the well-studied formalisms of the qualitative abstraction in QR is QDEs, which have been used by QSIM (Kuipers 1986, 1994) and Morven (Coghill 1996; Coghill and Chantler 1994).

**Fig. 3:**
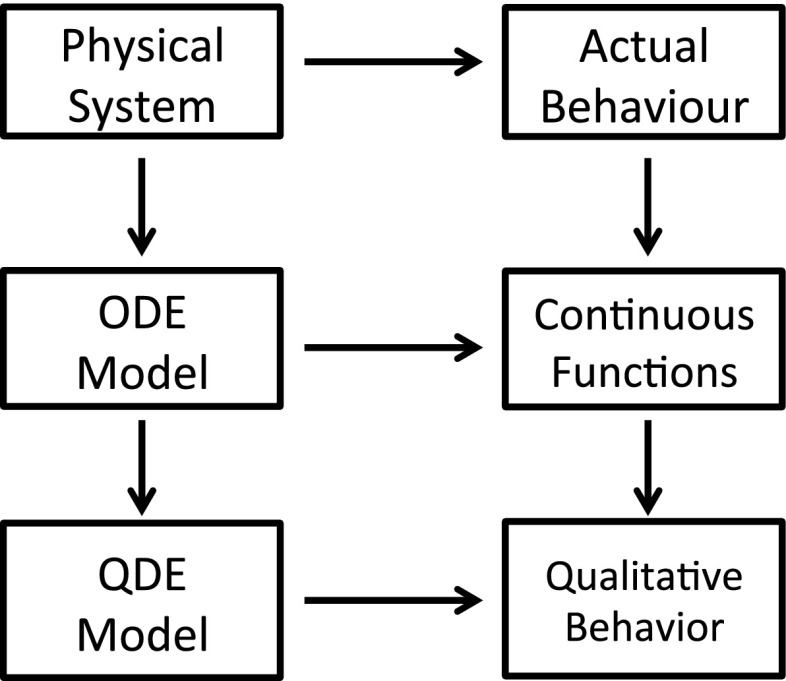
This diagram is obtained from Kuipers (1993). The *diagram* shows that all models are abstractions of the world. Qualitative models are related to ordinary differential equations, but are more expressive of incomplete knowledge

Ordinary differential equations (ODEs) quantitatively describe the behaviour of a dynamic system. A QDE model is an abstraction of a set of ODE models sharing the same model structure but with varying parameter values. Figure 3 shows the relationships between the physical systems, ODE models and QDE models in terms of quantitative and qualitative behaviour.

#### **Definition 1**

A *QDE* is a tuple of four elements, $\langle V, Q, C, T\rangle$, each of which is defined as follows (Kuipers 1994):

- $V$ is a set of *variables*, each of which is a ‘reasonable’ function of time.
- $Q$ is a set of *quantity spaces*, one for each variable in $V$.
- $C$ is a set of *constraints* applying to the variables in $V$. Each variable in $V$ must appear in some constraints.
- $T$ is a set of *transitions*, which are rules defining the boundary of the domain of applicability of the QDE.

From the above definition, we see that QDE is composed of several constraints, the so-called qualitative constraints, which restrict the generation of possible qualitative states. In addition, a quantity space is composed of several qualitative values that could be taken by a variable. In this research, we use QDEs to represent qualitative models, and for all variables we used the signs quantity space, which is composed of three values: *positive*, *zero* and *negative*.

### Synthetic models evaluation

Stepwise constructed qualitative models are simulated by a qualitative simulation engine, which produces a set of qualitative states. The given qualitative states of a target system are compared with the qualitative behaviour of synthetic models obtained from the evolutionary model construction process. The number of matched qualitative states between the target system and a synthetic model is recorded and taken as part of the fitness value for the model under evaluation. In this research, we use JMorven (Bruce and Coghill 2005) as the qualitative simulation engine.

#### Fitness function

The first evaluation method for composed models is to compare the qualitative states of the models with given data. A qualitative state for the evaluation purpose is the assignment of $N$ variables which appear in both the target system and composed model.

There could be $M$ qualitative states, and a vector is used to record each of these $M$ qualitative states. In this vector, each element will be the assignment of one variable. To evaluate a composed model, the following two sets will be compared element by element: one is the set of qualitative states generated by this composed model, and another is the given set of states demonstrated by the target system. In this way, a fitness value of a composed model is calculated by considering the overlapping part of the above two sets.

**Fig. 4:**
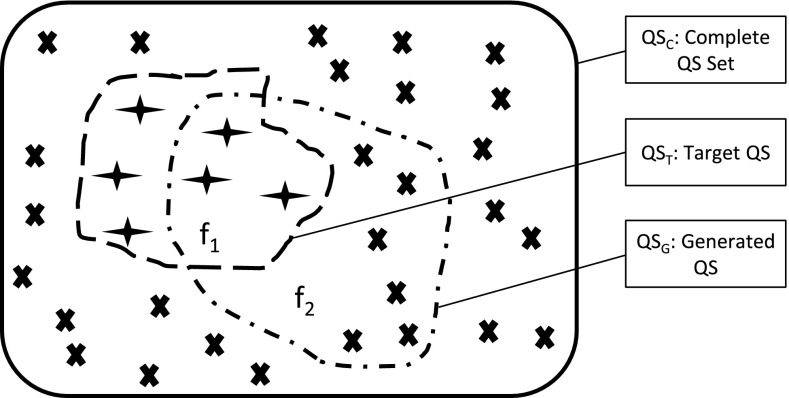
*Cross symbols in a frame with the solid line* indicate the set of complementary qualitative states, $QS_C$; *star symbols in a frame with dash line* show a set of given target qualitative states, $QS_T$; a composed model can produce a set of qualitative states, $QS_G$, which are in *a frame with the dash and dot line*; $f_1$ is the ratio of the $QS_T$ states which are also in $QS_G$ to all $QS_T$ states, and $f_2$ is the ratio of the $QS_C$ states which are also in $QS_G$ to all $QS_C$ states, as shown in Eqs. 4 and 5

Figure 4 shows that a synthetic model produces a set of qualitative states $QS_G$. A set of given target qualitative states $QS_T$ is compared with $QS_G$ to calculate the coverage of qualitative states in $f_1$. Another comparison is performed by comparing $QS_G$ with the set of all possible states $QS_C$, in which $f_2$ is the rate of matched qualitative states produced by the model under estimation. Therefore, the evaluation of a composed model can be achieved by jointly considering $f_1$ and $f_2$ in a fitness function F. Details of the calculation of $f_1$, $f_2$ and F are shown in Eqs. (4)–(6).

4 $$\begin{aligned} f_1&= \frac{\mid QS_G \cap QS_T \mid }{\mid QS_T \mid }\end{aligned}$$

5 $$\begin{aligned} f_2&= \frac{\mid QS_G \cap QS_C \mid }{\mid QS_C \mid }\end{aligned}$$

6 $$\begin{aligned} \mathrm{F}&= 1-\frac{1}{1+f_1+\frac{1}{1+f_2}} \end{aligned}$$

In the above Eqs. (4)–(6), ‘$|\cdot |$’ denotes the number of states in the set, $\mid QS_G \cap QS_C \mid$ indicates the set of overlapping states in both $QS_G$ and $QS_C$. Two qualitative states are the same if all their assignments of variables are the same.

The value of $f_1$ ranges from 0 (worst) to 1 (best), because as the more matched qualitative states between $QS_G$ and $QS_T$, the higher the value of $f_1$. The value of $f_2$ ranges from 0 (best) to 1 (worse), as the less matched spurious qualitative states between $QS_G$ and $QS_C$, the better the quality of the generated model. A fitness function F is summarised by standardising $f_1$ and $f_2$, and the value of F ranges from 0 (worst) to 1 (best).

Note that there could be different synthetic reactants in a composed model during the evolutionary modelling process because of the application of genetic addition and subtraction operators. In this work, we specify the compared reactants during the model evaluation process. Thus, we discard a composed model of which all reactants are not in the vector of variables for comparison with the target system.

#### Quantitative analysis of the structure of the synthetic models

The second evaluation method is to analyse the interactions among the reactants in the synthetic models. Here, the interactions are represented by connections in a model network consisting of arcs among biochemical substrates or their complex. In previous work (Brāzma et al. 1998; Gilbert et al. 2003; Wu et al. 2014), the structure of a synthetic model was evaluated quantitatively to investigate how many correct interactions among reactants in the generated models can be obtained.

In this research, two quantitative measures, *Compression* and *Coverage*, are employed to support the quantitative analysis of the quality of the composed models from qualitative modelling process. Both measures vary from 0 (worst) to 1 (best). If either the compression value or the coverage value is low in a particular model, it indicates that the structure of this generated model is very different from the target system, even if the qualitative states are covered at a high percentage.

*Compression* measures the percentage of matched common arcs between the target and generated models. Here, the arcs indicate interactions among biochemical reactants in a composed model. The Compression of a model is calculated as follows:

7 $$\begin{aligned} \mathrm{Compression} = \frac{|\mathrm{Intersection}|}{\mathrm{Max}(|\mathrm{Target}|, |\mathrm{Generated}|)} \end{aligned}$$

where $|\mathrm{Intersection}|$ represents number of matched arcs between the target system and generated model; the number of arcs in the target system is described as $|\mathrm{Target}|$; $|\mathrm{Generated}|$ denotes the number of arcs in the generated model; $\mathrm{Max}(|\mathrm{Target}|, |\mathrm{Generated}|)$ returns the bigger number of arcs between the target system and generated model.

*Coverage* calculates the percentage of matched arcs from the composed model in the target system. Details of the calculation are described as follows:

8 $$\begin{aligned} \mathrm{Coverage} = \frac{|\mathrm{Intersection}|}{|\mathrm{Target}|} \end{aligned}$$

where $|\mathrm{Intersection}|$ and $|\mathrm{Target}|$ are the number of matched arcs and numbers of arcs in the target system as defined above.

Considering the complicated issues in traditional modelling and analysis of biochemical systems, qualitative model evaluation focuses on the analysis of qualitative states of the system, without involving quantitative analysis in terms of the model structure. In this research, we include both qualitative and quantitative measurements to evaluate the learning results in terms of reactants’ qualitative states and interactions. This can offer a better evaluation of the feasibility and effectiveness of our proposed modelling methodology.

#### Components exploration based on Bayesian scores

Because of the characteristics of highly multimodal model space (Pang and Coghill 2010b), there may be many candidate models which can produce qualitative states covering the same given states. These models cannot be further discriminated, if we only consider fitness functions based on Eqs. (4)–(6). Therefore, we use Muggleton’s framework (1997) of learning from positive data to calculate the Bayesian score for each synthetic model with explored components. The Bayesian score is used to indicate the probability of the model being the true model during the QML process. The Bayesian score calculation is detailed in Pang and Coghill (2013) and briefly shown below:

9 $$\begin{aligned} \mathrm{Bayes}(M)=p \ln \frac{1}{g(M)}-\ln \mathrm{sz}(M) \end{aligned}$$

In Eq. (9), sz$(M)$ is the size of the given qualitative model M, $g(M)$ is the generality of the model, and $p$ is the number of positive examples. So the Bayesian scoring is the tradeoff between the size and generality of a model. Based on our previous work (Coghill et al. 2008), sz$(M)$ is estimated by summing up the sizes of all qualitative constraints in the model; $g(M)$ is defined as the proportion of qualitative states obtained from simulation to all possible qualitative states generated from given variables and their associated quantity spaces; $p$ is the number of given qualitative states. The bigger the Bayesian score of a candidate model, the higher the probability that this model is the correct model. In this research, explorations of components in synthetic models are guided by the above described Bayesian score, which has been incorporated into the SA fitness estimation process.

## Simulations and analysis

Three biochemical pathways are used as test cases for the evaluation of our proposed integrative modelling approach. The first pathway is the Ras/Raf-1/MEK/ERK signalling pathway (Yeung et al. 2000); the second one is the detoxification pathway (Ferguson et al. 1998) of Methylglyoxal; and the third one is the combinatorial stress response pathway of *Candida albicans* (Kaloriti et al. 2012). Qualitative states of these target pathways are abstracted from quantitative values obtained from simulators. Details of the pathway structures are retrieved from literatures as well as experiments.

Experiments on each test case is configured by the following settings: the total number of generations in the evolution process is 100; the number of evolved individual models in the population is 20 or 50; addition of one component is performed at each generation, subtraction of one component is carried out at every 10 generations, and crossing over two models is employed at each generation after the performance of addition or subtraction; each model seed in the initial population is an atomic component randomly selected from the component library.

The feasibility of our proposed stepwise QML method is firstly tested on a small scale of generations and populations on a computer with Intel Core 2 Duo CPU (2.4 GHz) and 4 GM memory. Therefore, the parameter settings of ES and SA are designed based on performance considerations and empirical selection, which has been tested individually. All the experiments have been performed for five trials for the RKIP pathway and ten trials for the MG-D pathways. It appeared that these parameter settings give the best results on a small scale.

Our next step is to investigate the effects of the ES and SA parameter settings on a large scale under the high-performance computing (HPC) environment. The results of simulations and analysis would present an overall influence of different algorithm parameters on the biochemical modelling issues.

### Biochemical pathways to be used for experiments

#### The RKIP inhibited ERK pathway

Signalling pathways play a pivotal role in many key cellular processes (Elliot and Elliot 2002). The abnormality of cell signalling can cause the uncontrollable division of cells, which may lead to cancer. The Ras/Raf-1/MEK/ERK signalling pathway (also called the ERK pathway) is one of the most important and intensively studied signalling pathways, which transfers the mitogenic signals from the cell membrane to the nucleus (Yeung et al. 2000). It is de-regulated in various diseases, ranging from cancer to immunological, inflammatory and degenerative syndromes, and thus represents an important drug target. Ras is activated by an external stimulus, via one of many growth factor receptors; it then binds to and activates Raf-1 to become Raf-1*, or activated Raf, which in turn activates MAPK/ERK Kinase (MEK) which in turn activates Extracellular signal Regulated Kinase (ERK). This cascade (Raf-1 $\rightarrow$ Raf-1* $\rightarrow$ MEK $\rightarrow$ ERK) of protein interaction controls cell differentiation with the effect being dependent upon the activity of ERK. RKIP inhibits the activation of Raf-1 by binding to it, disrupting the interaction between Raf-1 and MEK, thus playing a part in regulating the activity of the ERK pathway.

**Fig. 5:**
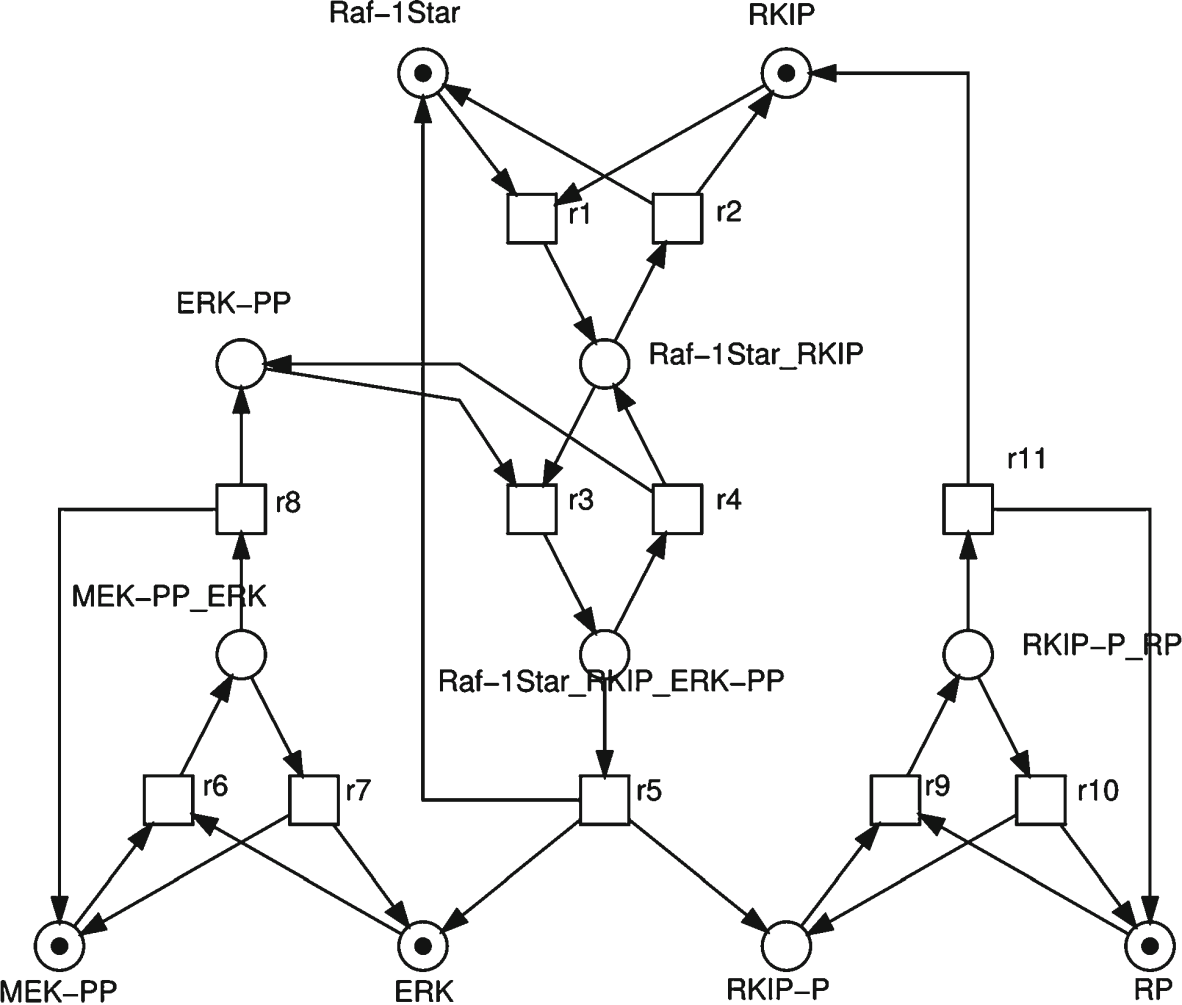
A graphical representation of the ERK signalling pathway regulated by RKIP

A number of computational models have been developed to understand the role of RKIP in the pathway and ultimately develop new therapies (Cho et al. 2003; Calder et al. 2004). In this research, we use the RKIP inhibited ERK pathway (called RKIP pathway in this research) as described in Cho et al. (2003) to test our proposed modelling approach. Figure 5 shows a representation of the ERK signalling pathway regulated by RKIP.

#### The detoxification pathway of MG

Cell death is related to the excessive production of Methylglyoxal (MG), a naturally occurring toxic electrophile which is harmful to cells (Cooper 1984). A detoxification pathway of MG (MG-D) exists in many diverse groups of organisms, for instance, human, mouse, yeast, and fungi, for the protection from the toxic effect of MG (Ferguson et al. 1998). The MG detoxification pathway is of interest to both biologists and physicists. Currently, research into this pathway is still ongoing and carried out as a systems biology project, in which both biological experimentalists and modellers are involved (Pang and Coghill 2011).

**Fig. 6:**
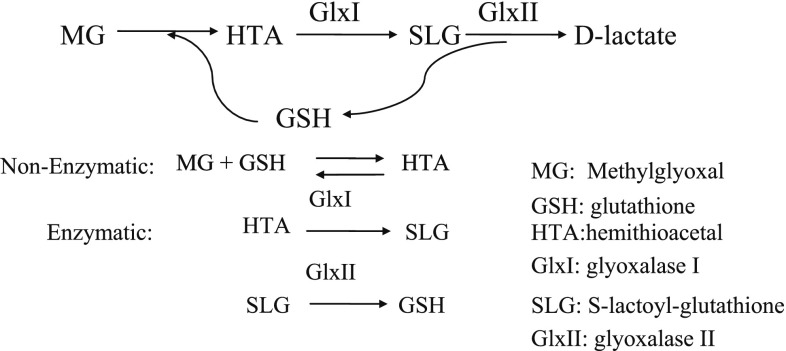
A graphical presentation of the detoxification pathway of MG, obtained from Pang and Coghill (2011)

Figure 6 shows the current understanding of the MG-D pathway. A large quantity of MG is outside the cell and MG can easily cross the cell membrane and enter the cell, in which glutathione (GSH) reacts spontaneously with MG to produce hemithioacetal (HTA). HTA is catalysed by the enzyme glyoxalase I (GlxI) and converted into S-lactoyl-glutathione (SLG). Then, the SLG is catalysed by a second enzyme glyoxalase II (GlxII) and converted into GSH and non-toxic d-lactate. There are one non-enzymatic biochemical reactions and two enzymatic reactions in the whole pathway. Note that the non-toxic d-lactate is not included in this pathway, as it does not contribute to the kinetics of the pathway.

#### The combinatorial stress pathway of *Candida albicans*

*Candida albicans* (*C. albicans*) is one of the major fungal pathogens of humans. There exists a range of environmental stresses in the hosts of *C. albicans* and the most common stresses are osmotic, oxidative, and nitrosative stresses. *C. albicans* is exposed to these stresses and adapts itself by responding to the stress conditions. In the wild, *C. albicans* cells are frequently exposed simultaneously to combinations of these stresses and yet the effects of such combinatorial stresses have not been explored (Kaloriti et al. 2012).

Therefore, it is crucial to set up a modelling platform in silico to investigate the combinatorial stress responses in *C. albicans* using qualitative biochemical models, considering the facts that data about stress responses are very sparse (especially those for nitrosative stress). Our aims are not only to verify biochemical interactions from the wet-lab, but also to discover potential reactions which could inhibit or increase the growth of *C. albicans* under the condition of combinatorial stresses.

**Fig. 7:**
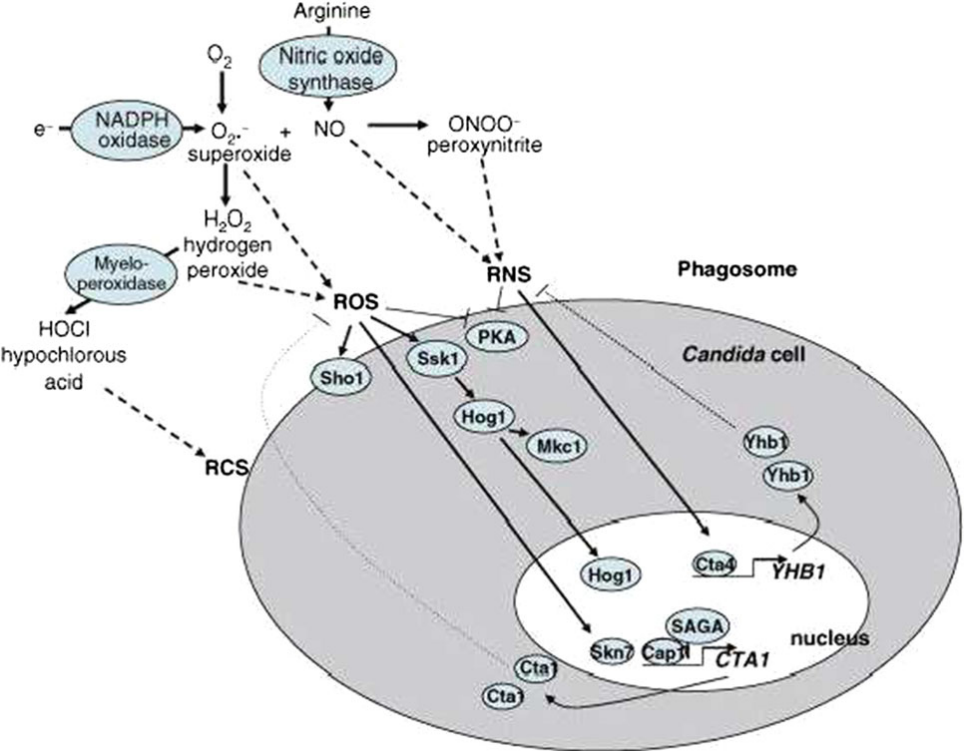
A graphical presentation of the pathways involved in oxidative and nitrosative stress responses in *C. albicans*, obtained from Brown et al. (2009)

Figure 7 shows a graphical presentation of the proteins and pathways involved in the oxidative and nitrosative stress responses in *C. albicans* (Brown et al. 2009). In this research, components involving reactants in *C. albicans* can be explored by the integrative modelling approach, so that predictions of key response pathways to the combinatorial stresses could be made.

### Fitness evaluation of composed models

**Fig. 8:**
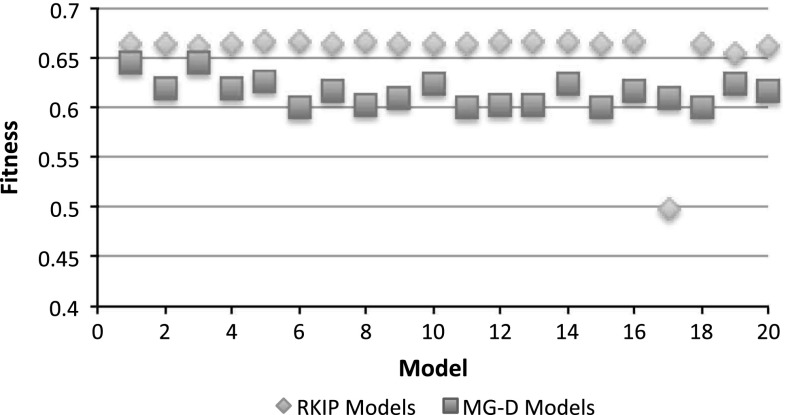
Fitness values of the models produced and learned by the stepwise model learning (colour figure online)

According to the fitness evaluation function given in Eq. (6), fitness value ranges from 0 (worst) to 1 (best). At the end of evolutionary learning process, there is a set of evolved candidate models with best fitness values. Figure 8 shows two sets of fitness values of developed models for the RKIP and MG-D pathways.

In this work, a high fitness value means a good coverage of qualitative states produced by the corresponding candidate model. The blue diamond and red square shaped dots indicate fitness values of 20 synthetic models of the RKIP and MG-D pathways, respectively. Most fitness values of the composed RKIP models are around 0.65, and the ones of the constructed MG-D models are between 0.6 and 0.65. Since the model development process starts from a set of simple components and is driven in a stepwise manner, the generation of models with high fitness values indicates that our stepwise QML methodology is feasible to infer desired models of the target biochemical pathways based on qualitative data and incomplete knowledge.

### Topology analysis of explored models

#### Generation of target reactants

A general principle for analysing the topologies of generated models is to investigate how many essential reactants can be generated. Essential reactants in a target pathway can be obtained through composition of atomic components, thus observable elements in experimental environment can be mapped to these essential reactants in the synthetic models. Changes of concentrations of the reactants in wet-lab can be analysed qualitatively and simulated quantitatively in silico. In our study, structures of synthetic models vary from target biochemical pathways because of the stepwise composition of components. Regarding the aims of generating interest reactants and hidden complexes, different interactions between reactants in the models can be preserved for further biochemical investigation.

Table 2 shows a comparison of generated reactants between a synthetic RKIP model with the highest fitness value (0.6665) and the target RKIP pathway. There are 11 reactants (from No.1 to No.11) in the target RKIP pathway, of which 7 reactants are obtained in the synthetic RKIP model. The missing reactants in the synthetic model are complex reactants in the target RKIP pathway. Thus, we can conclude that our stepwise qualitative model learning strategy can drive the model development process towards the generation of essential reactants in a target biochemical pathway.

**Table 2:** Generation of essential reactants in a stepwise composed RKIP model

No.	Reactant	Model	RKIP
1	Raf1	$\surd$	$\surd$
2	RKIP	$\surd$	$\surd$
3	Raf1$|$RKIP		$\surd$
4	Raf1$|$RKIP$|$ERKPP		$\surd$
5	ERK	$\surd$	$\surd$
6	RKIPP	$\surd$	$\surd$
7	MEKPP	$\surd$	$\surd$
8	MEKPP$|$ERK		$\surd$
9	ERKPP	$\surd$	$\surd$
10	RP	$\surd$	$\surd$
11	RKIPP$|$RP		$\surd$
12	MEKPP$|$RP	$\surd$	

One complex reactant generated in the synthetic model (No.12, MEKPP$|$RKIPP) does not exist in the target RKIP pathway. MEKPP$|$RKIPP indicates a potential interaction between MEKPP and RKIPP in the synthetic model. This plausible interaction is actually supported by tracking the reachable path in the RKIP pathway:

$$\begin{aligned}&\mathrm{RKIPP} \rightarrow \mathrm{RKIPP}|\mathrm{RP} \rightarrow \mathrm{RKIP}\rightarrow \mathrm{RKIP}|\mathrm{Raf1} \\&\quad \rightarrow \mathrm{ERKPP}|\mathrm{RKIP}|\mathrm{Raf1} \rightarrow \mathrm{ERK}\\&\quad \rightarrow \mathrm{ERK}|\mathrm{MEKPP} \rightarrow \mathrm{MEKPP}. \end{aligned}$$

Therefore, one conclusion in this research is that alternative interactions among reactants can be explored by our proposed methodology.

There are six reactants (M, G, H, S, G1 and G2) in the MG-D pathway, and qualitative states containing the values of M, G, H, and S are used for model evaluations. Table 3 presents reactants of a synthetic model with the highest fitness value (0.6452). All the reactants in the target MG-D pathway are generated and one more complex (No.7, G$|$G2) is produced. Although the topology of the synthetic model is different from the target one, the aim of learning a biochemical pathway from qualitative states is achieved. In addition, up till now the MG-D pathway is not fully understood. This means the composed model may suggest interesting biological experiments in the future.

**Table 3:** Generation of essential reactants in a stepwise composed MG-D model

No.	Reactant	Model	MG-D
1	M	$\surd$	$\surd$
2	G	$\surd$	$\surd$
3	H	$\surd$	$\surd$
4	S	$\surd$	$\surd$
5	G1	$\surd$	$\surd$
6	G2	$\surd$	$\surd$
7	G$|$G2	$\surd$	

#### Generation of target interactions

After models are composed, essential reactions existing in the target biochemical system should be obtained from these synthetic models. Thus, it is important to evaluate how many target interactions in the target system are obtained in these constructed models. Two measures are used, Compression and Coverage, as introduced in Sect. 3.3.2 and the interaction information of reactants in the composed RKIP and MG-D models is analysed by these two measures. Details are given as follows.

**Fig. 9:**
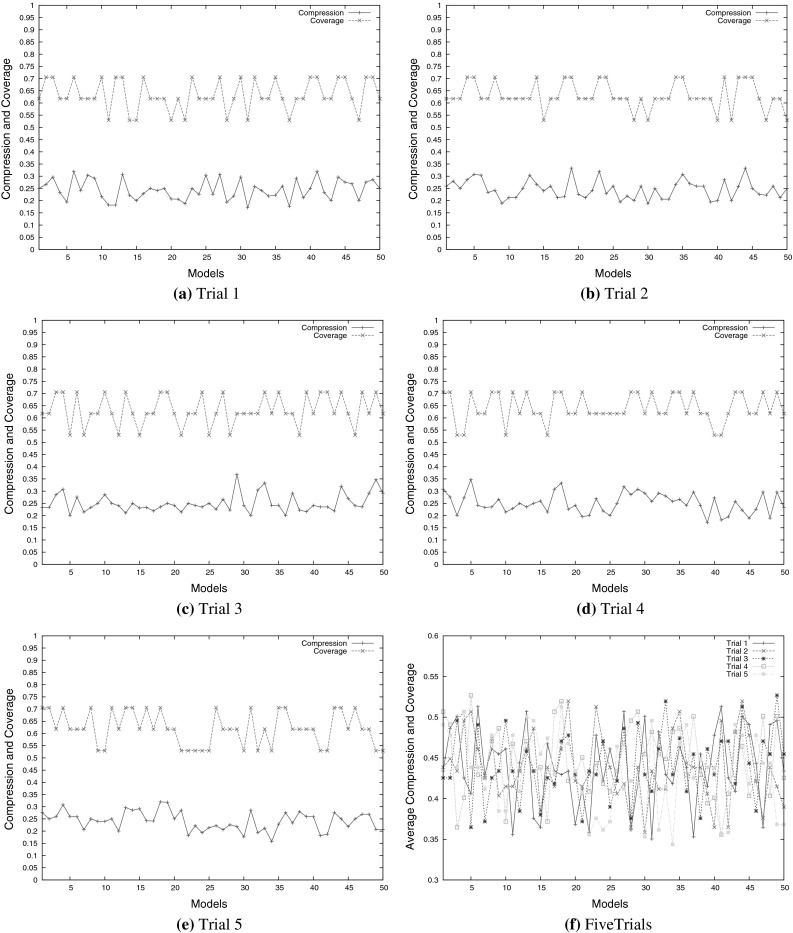
Compression and coverage of the synthetic RKIP models in five trials. **a** Trial 1, **b** trial 2, **c** trial 3, **d** trial 4, **e** trial 5, **f** five trials

Figure 9 shows the generation of RKIP models. From this figure, one can see that most of the coverage values generated from the five trials are between 0.5 and 0.7, which indicates that up to 8 reactions (11 reactions in total) are obtained. These high coverage values support the conclusion in Sect. 4.3.1 that our proposed modelling methodology can generate most of the target biochemical reactants in the synthetic models.

In addition, most of the compression values for the RKIP models composed in these five trials are between 0.15 and 0.35,which indicates that there are many non-target reactions generated in the constructed RKIP models. These suggested reactions (interactions among biochemical reactants) are potential biochemical signalling routes, which may help biologists to further investigate the RKIP pathway.

Figure 9f shows the average values of compression and coverage for each model in all five trials. Most of the average values are between 0.35 and 0.55. Regarding the high compression values of these models, one can see that the synthetic RKIP models can be different from the given target RKIP pathway in terms of topology (interactions among substrates).

In this research, the MG-D pathway is reconstructed by applying composition rules to explore partial modules of the given pathway. After ten trials of simulations, the labels of complex in composed models are formed by joining labels of reactants from the modelling seeds and added components. Therefore, the joined and formed complex can be renamed for comparison between the model and target pathways. For instance, complex ‘H’ in a reaction ‘$M+G \rightarrow H$’ can be renamed as ‘G$|$M’ to represent the complex formed from ‘M’ and ‘G’. Thus, the target MG-D pathway reaction information is renamed for compression and coverage analysis as follows: ‘H’ is renamed as ‘G$|$M’, ‘S’ is renamed as ‘G1$|$H’, and ‘G’ is renamed as ‘G2$|$S’.

**Fig. 10:**
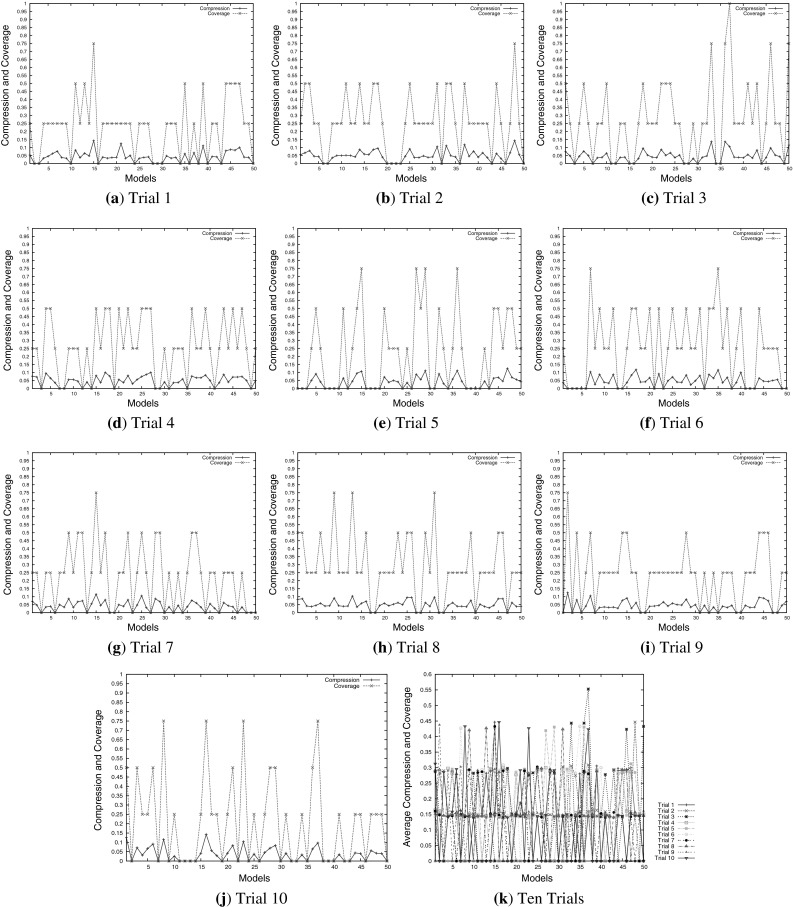
Compression and coverage of the synthetic MG-D models in ten trials. **a** Trial 1, **b** trial 2, **c** trial 3, **d** trial 4, **e** trial 5, **f** trial 6, **g** trial 7, **h** trial 8, **i** trial 9, **j** trial 10, **k** ten trials

Figure 10 shows the generation of MG-D pathways. From this figure, one can see that most coverage values for these models are between 0 and 0.75, and the coverage value of one model from trial 3 in Fig. 10c is equal to 1, which means all the given reactions are obtained. Moreover, most compression values of the MG-D models are between 0 and 0.15, which indicates that there are lots of non-target reactions generated in the constructed models. Figure 10k presents the average values of compression and coverage for each model in all ten trials. Most of average values are between 0 and 0.55, suggesting that these synthetic MG-D models are very different from the given target pathway in terms of structure.

### Explored interactions in developed models

We apply the integrative top-down and bottom-up modelling approach to explore key and potential interactions in the synthetic models of the combinatorial stress response pathway in *C. albicans*. Explored interactions represent biochemical reactions and indicate effects of reactants inhibiting or increasing productions of other reactions in the models. Thus, potential biochemical links can be established using our integrative modelling approach, and these links will provide guidance for future investigation by biologists.

Table 4 shows the top 10 % of the most frequently explored components in the developed 20 models of *C. albicans*. The numbers in the frequency column indicate the frequencies of corresponding components explored from these synthetic models. If a component is selected and integrated in models more frequently during the modelling process, we can consider the component as an important interaction among reactants for the biochemical model under construction. A component is an actual interaction among reactants denoted by abbreviated compound names of *C. albicans*. For instance, the most selected component ‘$\mathrm{Cap1Ox,\,PBS2 }\rightarrow \mathrm{Cap1Ox}|\mathrm{PBS2}$’ illustrates that a complex $\mathrm{Cap1Ox}|\mathrm{PBS2}$ is made from $\mathrm{Cap1Ox}$ and $\mathrm{PBS2}$ in a biochemical reaction. Possible interactions among these three reactants can be examined by biological experiments and further biochemical relationships may be discovered for the target system.

**Table 4:** Explored components with frequency from modelling *C. albicans*

Frequency	Explored Components	Frequency	Explored components
109	$\mathrm{Cap1Ox},\mathrm{PBS2} \rightarrow \mathrm{Cap1Ox}|\mathrm{PBS2}$	71	$\mathrm{Ssk2} \rightarrow \mathrm{Glr1},\mathrm{Cap1Ox}$
93	$\mathrm{GPX1} \rightarrow \mathrm{Pbs2PP},\mathrm{CAP1}$	70	$\mathrm{Tsa1Ox} \rightarrow \mathrm{Ssk2},\mathrm{Cap1Ox}$
91	$\mathrm{Trx1Red,\,PBS2} \rightarrow \mathrm{PBS2}|\mathrm{Trx1Red}$	70	$\mathrm{SSK2} \rightarrow \mathrm{Pbs2PP,\,GLR1}$
81	$\mathrm{GPX1} \rightarrow \mathrm{Cap1Ox,\,Trr1Ox}$	70	$\mathrm{Hog1Red,\,CTA1} \rightarrow \mathrm{CTA1}|\mathrm{Hog1Red}$
74	$\mathrm{H2I2In} \rightarrow \mathrm{Glr1,\,Pbs2PP}$	70	$\mathrm{GSH,\,Hog1Red} \rightarrow \mathrm{PBS2}$
73	$\mathrm{Tsa1Ox,\,PBS2} \rightarrow \mathrm{PBS2}|\mathrm{Tsa1Ox}$	70	$\mathrm{Cap1Ox,\,GRXOx} \rightarrow \mathrm{Cta1}$
72	$\mathrm{PBS2}|\mathrm{Tsa1Ox,\,GSSG}$ $\rightarrow \mathrm{GSSG}|\mathrm{PBS2}|\mathrm{Tsa1Ox}$	70	$\mathrm{Cap1Ox,\,CAP1} \rightarrow \mathrm{CAP1}|\mathrm{Cap1Ox}$

The models of the *C. albicans* stress response pathway are initialized and developed independently by our proposed modelling approach in a heuristic manner. Although models are explored by adding different components from the component library, models containing these components with high frequency are heuristically explored and easily accepted by the bottom-up approach. Thus, these ‘hot’ components could be the key and potential interactions in the pathway in the sense that they may play important roles in the stress response and adaptation. Further investigations of biochemical functions of these hot components can be carried out by biologists.

## Conclusions

It is always interesting for life scientists to obtain alternative solutions (different topologies of biochemical systems) that may not be discovered in nature, thus the exploration of the topological landscape of the biochemical systems is very important. It is also useful for synthetic biologists to construct novel designs for desired behaviour of biochemical systems in silico before carrying out experimental work.

In this research, we show how general biochemical systems can be modelled and evolved in an automatic manner by reusing, composing, and evolving biochemical modular components, based on an integrative top-down and bottom-up QML approach. Two main aims of learning biochemical systems are achieved in this research: the first is to learn reactants in a given biochemical pathway by analysing the observed qualitative states; another aim is to evolutionarily explore plausible structures of a target pathway at a qualitative level. As a result, we propose an integrated qualitative model learning framework in the presence of incomplete knowledge and qualitative data. Our approach can be used in both the context of computational systems biology for model construction and synthetic biology for the modular design of biochemical systems.

In future research, we will investigate the way of constructing ad hoc component library for the pathway of interest, for instance, metabolic or signalling pathways in a particular species. One way to achieve this is to take the functionally similar or equivalent pathways in model organisms as input and try to evolve components from these pathways. In addition, we are interested in investigating the structural differences of functionally equivalent components of a pathway across different species, especially how these structural differences could be analysed in silico through our QML approach. To achieve this key aim, reliable parts of biochemical systems should be identified and preserved in the component library, and these parts should be further verified by experiments and available data. Then, more details about biochemical systems can be obtained by inferring possible or undiscovered reactants and interactions during the composition process. Finally, to deal with varying data availability, we will explore the following two strands: first, based on our previous study (Coghill et al. 2008; Pang and Coghill 2007), we will consider the situation where there are very few qualitative states available for learning; second, if more biological data could be obtained, we would like to explore the use of semi-quantitative model learning approaches (Vatcheva et al. 2006) to precisely study the parameters of biochemical systems and how semi-quantitative approaches can complement our QML framework.
